# Mental health of israeli employees with autism spectrum disorders following COVID-19-related changes in employment status

**DOI:** 10.1192/j.eurpsy.2021.719

**Published:** 2021-08-13

**Authors:** Y. Goldfarb, E. Gal, O. Golan

**Affiliations:** 1 Occupational Therapy, University of Haifa, Haifa, Israel; 2 Psychology, Bar Ilan University, Ramat Gan, Israel; 3 Psychiatry, University of Cambridge, Cambridge, United Kingdom

**Keywords:** Adults with ASD, Employment, COVID-19, Mental health

## Abstract

**Introduction:**

The COVID-19 pandemic caused employment related challenges worldwide. Adults diagnosed with Autism Spectrum Disorders (ASD) are especially vulnerable, due to pre-existing employment challenges, intolerance to changes and uncertainty and high levels of related anxiety.

**Objectives:**

To examine COVID-19 related changes in work experiences and mental health of employees with ASD who held a steady job before the COVID-19 outbreak.

**Methods:**

Data were collected from 23 participants diagnosed with ASD (4 females), aged 20–49, who answered an online administered survey at two timepoints: prior to the COVID-19 outbreak, and during the outbreak. Self-reports included measures of background and employment status; mental health (General Health Questionnaire-12); job satisfaction (Minnesota Satisfaction Questionnaire); and satisfaction of psychological needs at work (Psychological Need Satisfaction and Frustration – Work domain).

**Results:**

Participants who continued to physically attend work maintained pre-COVID-19 levels on all assessed variables. Participants who transitioned to remote work from home preserved their salary levels and job satisfaction, but showed a marginally significant deterioration in mental health and a significant decrease in the satisfaction of their needs for competence and autonomy at work. Unemployed participants showed a significant decrease in mental health.

**Conclusions:**

Results highlight employment as a protective factor from the potential negative implications of COVID-19 on mental-health of employees with ASD. Employees who transition to working from home require personalized work-support plans due to the possible negative effects of this transition on mental health. Maintaining the routine of physically reporting to work should be preferred, when possible.

